# Supplementary material for: Effective design and inference for cell sorting and sequencing based massively parallel reporter assays
Source: Bioinformatics. 2023 Apr 21;39(5):btad277. doi: 10.1093/bioinformatics/btad277 (PMC10182853; doi:10.1093/bioinformatics/btad277)
Supplement: btad277_Supplementary_Data [file btad277_supplementary_data.pdf]

Supplementary Information: Effective design and inference for cell sorting and sequencing based massively parallel reporter assays

Pierre-Aurélien Gilliot[1] **and Thomas E. Gorochowski**[1,2]

[1]**School of Biological Sciences, University of Bristol, 24 Tyndall Avenue, Bristol, BS8 1TQ, UK**
[2]**BrisEngBio, School of Chemistry, University of Bristol, Cantock's Close, Bristol, BS8 1TS, UK**

CONTENTS

Supplementary Notes **2**
Supplementary Note 1: Comparing experimental designs using Bayesian decision theory 2
Supplementary Figures **4**
Supplementary Figure 1: A staircase effect results when a few sorting bins are used 4
Supplementary Figure 2: Sampling variation between the MOM and ML inference methods . . 5
Supplementary Figure 3: Comparing the accuracy of the estimators 6
Supplementary Figure 4: Comparing the estimators across an entire genetic library 7
Supplementary Figure 5: Coverage rate for the ML inference method 8
Supplementary Figure 6: Scalability of the parallelized ML estimator 9
Supplementary Figure 7: Differences in inference accuracy 10
Supplementary Figure 8: Optimal number of cells and sequencing reads when using MOM . . 11
Supplementary Figure 9: Optimal number of cells and sequencing reads when using ML . . . 12
Supplementary Figure 10: Optimal number of bins for cell sorting when using MOM and ML . 13
Supplementary Figure 11: Evaluating the hybrid CNN+RNN neural network performance . . . 14
Supplementary Figure 12: Accuracy limits for models trained on the Cambray *et al.* data set. 15
References **16**

Supplementary Note 1: Comparing experimental designs using Bayesian decision theory

Here we derive the criterion used to compare different Flow-seq experimental design. Our goal is to estimate for each genetic variant the corresponding fluorescence parameters $\theta^* \in \mathbb{R}^2$ (either the Log-Normal or Gamma parameters). For this purpose, the Flow-seq protocol is used, which depends on a choice of experimental factors $e \in \mathbb{R}^3$, which includes 1. the number of sequencing reads, 2. the number of cells sorted, and 3. number of bins used for cell sorting.

The outcome for the Flow-seq experiment is sequencing data that acts an observable $z \sim P(z \mid \theta^*; e)$. This data is then processed by an estimator (either ML or MOM) to claculate the fluorescence parameters $\theta \sim P(\theta \mid z)$ for each genetic variant. $P(z \mid \theta^*; e)$ accounts for the randomness that occurs when growing the cells containing the library and during the sequencing process, while $P(\theta \mid z)$ describes the randomness occurring during the Nelder-Mead optimization step used by the ML estimator. However, we found this second source of randomness negligible during our experiments and so neglected its contribution.

For different values of experimental factors e, the deviations from the true value θ^* will be most important and can be measured using an appropriate loss function. A popular choice for experimental design involves minimizing the total parameter variance of the estimates (i.e., A-design[1]), although such a choice would ignore the bias of the estimates[2]. To better understand the influence of the experimental design choices e on the fluorescence parameter estimates, we considered two different loss functions. Firstly, a loss function comparing globally the inferred and ground-truth distribution, as measured by the 1-Wasserstein distance:

$$L(\theta, \theta^*) = \int_{\mathbb{R}} \mid F_\theta(x) - F_{\theta^*}(x) \mid dx,$$

with $F(x)$ being the respective cumulative distribution function. And secondly, a local loss function to quantify the differential error between the two parameters of the fluorescence distribution $\theta = (\theta_1, \theta_2)$:

$$L(\theta_i, \theta_i^*) = \frac{\mid \theta_i - \theta_i^* \mid}{\theta_i^*}, \quad i = 1, 2.$$

The choice of the absolute relative error loss instead of the more common squared error loss was motivated for two reasons. Firstly, the logarithmic scaling used in flow cytometry binning will inherently lead to a decrease in measurement quality for constructs displaying high-fluorescence. This would inflate the loss if using the squared error. Secondly, the absolute error loss is more interpretable and useful for biological engineers, who prefer to bound the magnitudes of the error rather than the absolute value[3].

Utility functions are often impossible to minimize uniformly with respect to the decision (here the experimental design e) as the parameter θ^* is unknown and the inferred parameter θ is a random variable. A frequentist approach would imply averaging over all replicates of the Flow-seq experiments to calculate the *frequentist risk*[4]:

$$R(\theta^*, e) = \mathbb{E}_{z \sim P(z \mid \theta^*; e)}[L(\theta, \theta^*)].$$

However, the frequentist risk is a function of the parameter θ to estimate, which is highly variable in these types of experiments.

To resolve this issue, we can define the *integrated risk* $r(e)$, which considers the average over a prior

$\pi(\theta^*)$ of the parameter θ^*:

$$r(e) = \mathbb{E}_{\theta^* \sim \pi(\theta^*)}[R(\theta^*, e)].$$

The integrating risk induces a total ordering, allowing us to compare the loss function for different experimental designs and inference methods. We resorted to Monte-carlo simulations to compute the integrated risk as it contains a double integral over the parameter and the observations space, which is challenging to compute analytically. This lead us in practice to averaging the inference results over a library of constructs (either from the Taniguchi[5] or Cambray[6] data sets), which constitutes our prior on the parameter space, and conducted many Flow-seq replicates to average over the Flow-seq realisations.

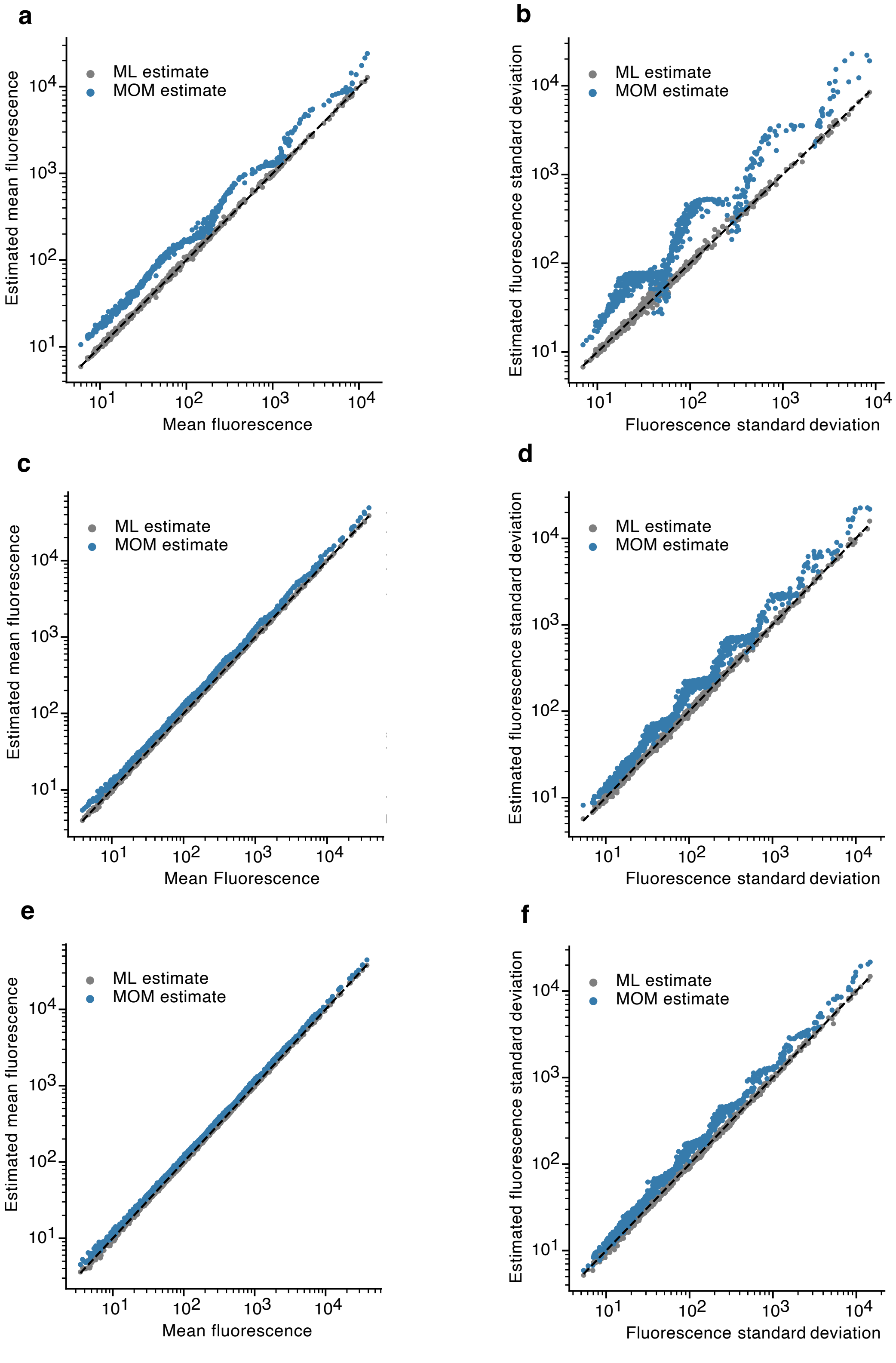

Supplementary Figure 1: A staircase effect results when a few sorting bins are used. Estimates for the fluorescence mean (left panels) and standard deviation (right panels) are compared with the ground truth when using an increasing number of sorting bins. Each point corresponds to a genetic variant colored blue for the Method of Moments (MOM) estimator, and grey for the Maximum Likelihood (ML) estimator. The black dashed line shows $y = x$ (i.e., a perfect estimation). (**a, b**) Cells are sorted into 6 bins (**c, d**) Cells are sorted into 8 bins. (**e, f**) Cells are sorted into 12 bins.

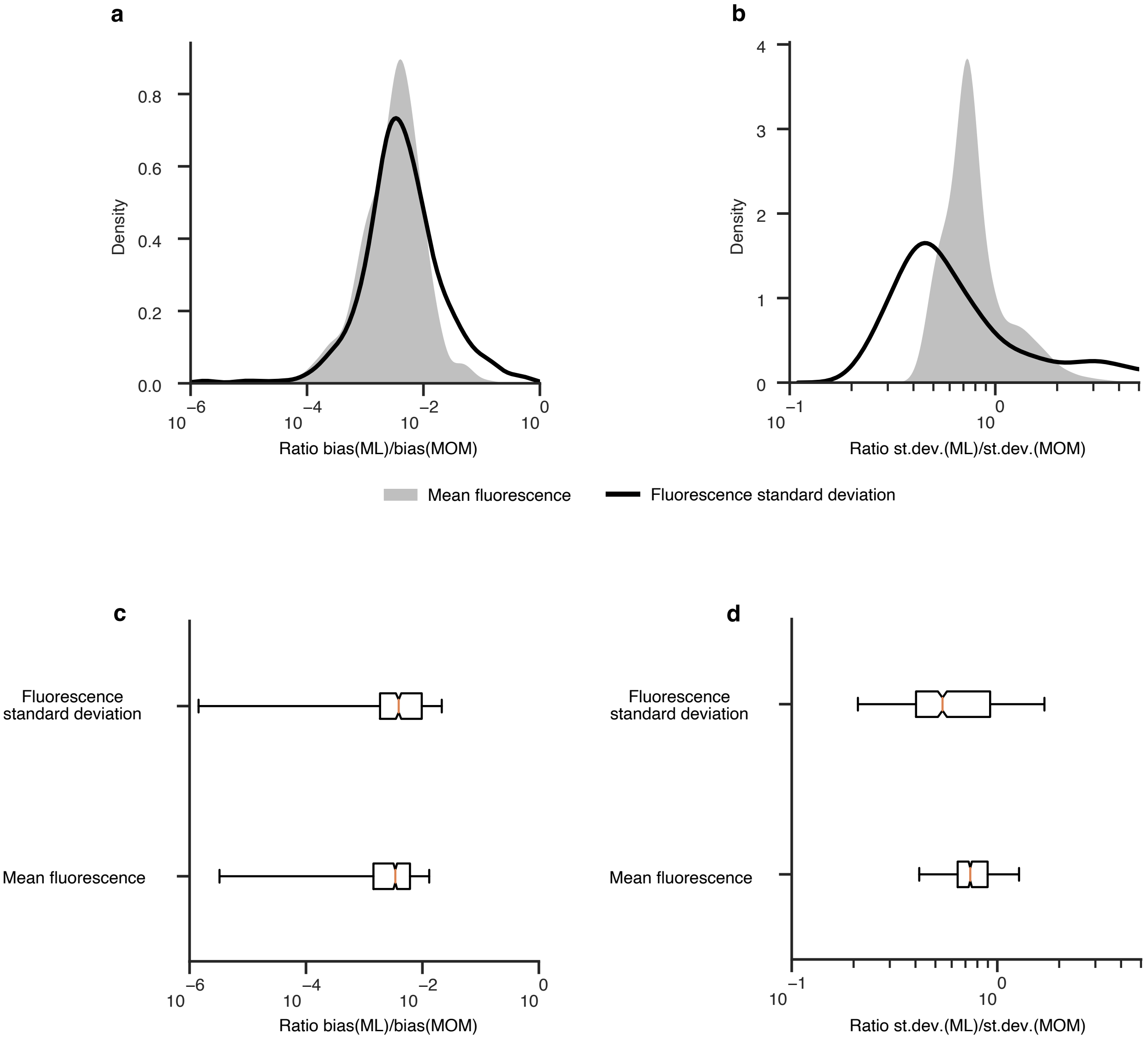

Supplementary Figure 2: Sampling variation between the MOM and ML inference methods. Sampling variation was estimated by carrying out 500 synthetic Flow-seq simulations using biologically realistic expression characteristics. (**a**) Kernel density estimation of the ratio between the bias of the ML estimator and the bias of the MOM estimator when estimating the fluorescence mean or standard deviation across simulations. (**b**) Kernel density estimation of the ratio between the standard deviation of the ML estimator and the standard deviation of the MOM estimator when estimating the fluorescence mean or standard deviation across simulations. (**c**) Box plot summarising the data shown in panel a. (**d**) Box plot summarising the data in panel c.

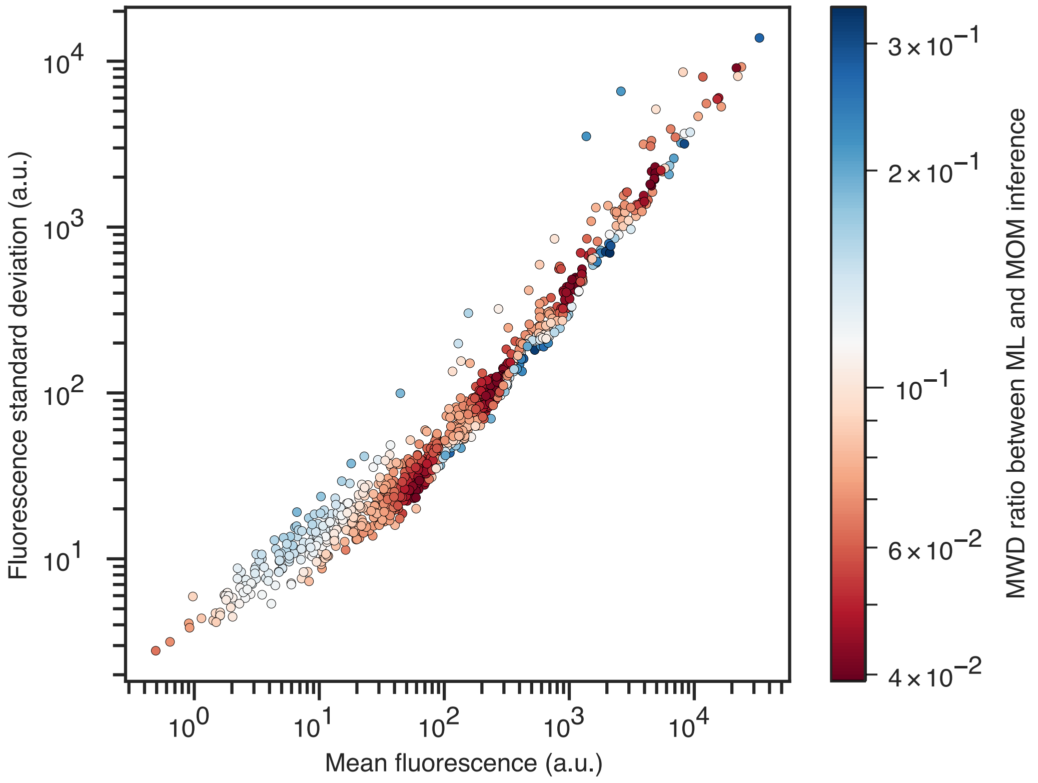

Supplementary Figure 3: Comparing the accuracy of the estimators. ML estimator outperforms the MOM estimator in most cases. Each circle represents a genetic construct, colored according to the Mean Wasserstein Distance (MWD) ratio between ML and MOM inference. The fluorescence mean and fluorescence standard deviation of each construct were used as coordinates for plotting this ratio. In all cases, this ratio is smaller than 1 (maximum at 0.34), indicating the superior performance of the ML method compared to the MOM method. Each genetic variant was characterized using a simulated Flow-seq experiment with 1018 genetic variants, 10^7 sequencing reads, 10^6 cells sorted, a PCR amplification factor of 10^2, 8 bins for cell sorting, and a maximum fluorescence of $f_{max} = 10^5$, using the fluorescence distribution parameters from the Taniguchi library. 500 different replicate simulations were generated to compute the mean 1-Wassertein distance between the inferred and ground-truth distribution when using ML and MOM inference.

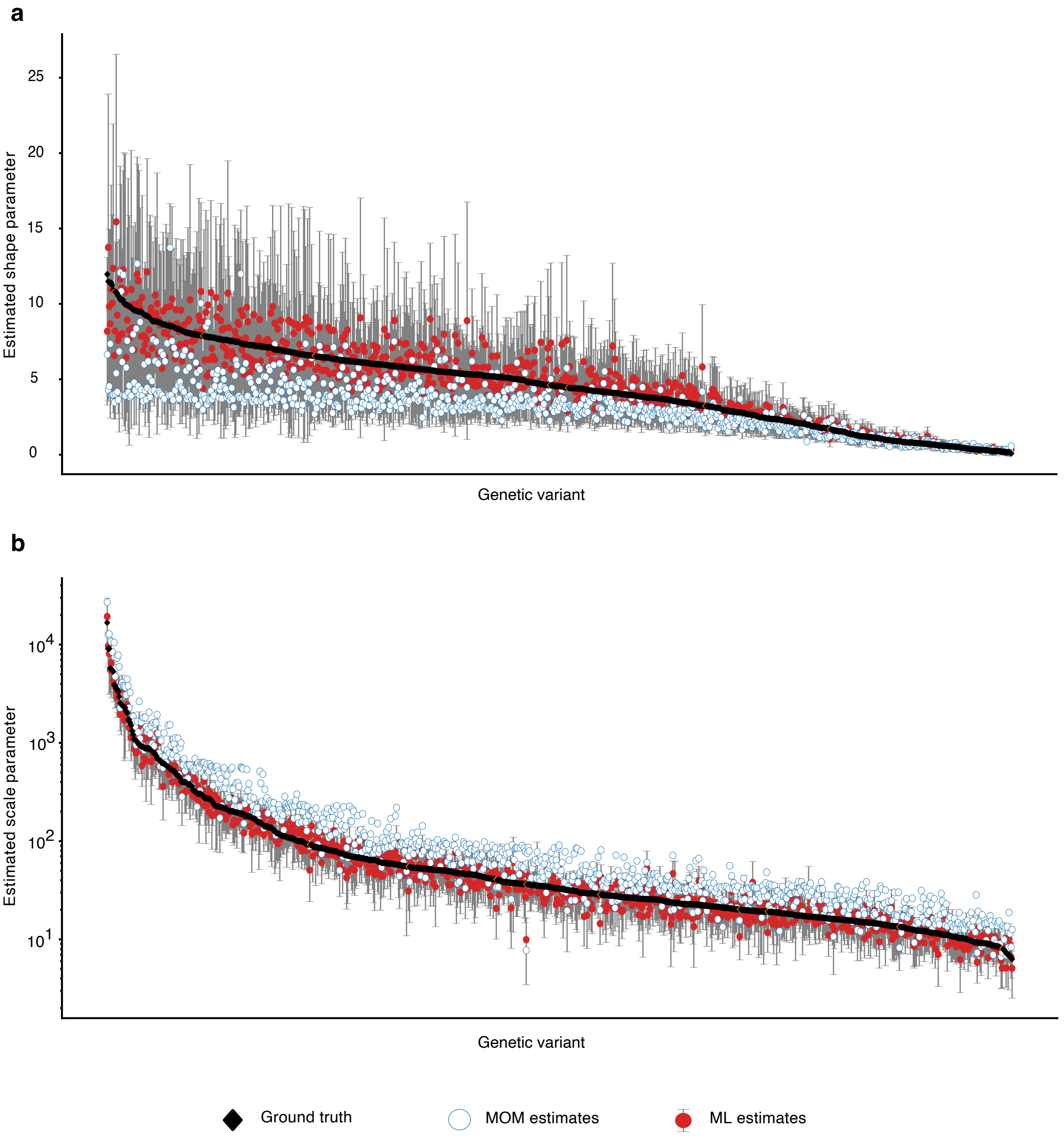

Supplementary Figure 4: Comparing the estimators across an entire genetic library. Estimated shape parameter (**a**) and estimated scale parameter (**b**) for the Gamma distribution of each genetic variant. Each genetic variant was characterized using a simulated Flow-seq experiment with 1018 genetic variants, 10^7 sequencing reads, 10^6 cells sorted, a PCR amplification factor of 10^2, 8 bins for cell sorting, and a maximum fluorescence of $f_{max} = 10^5$, using the fluorescence distribution parameters from the Taniguchi library. Points show the inferred parameter value for the MOM (blue outline, white centre) and ML (red filled) methods, with the ground truth shown by a black-filled diamond. For the ML values, error bars show the 95% confidence interval for the estimate.

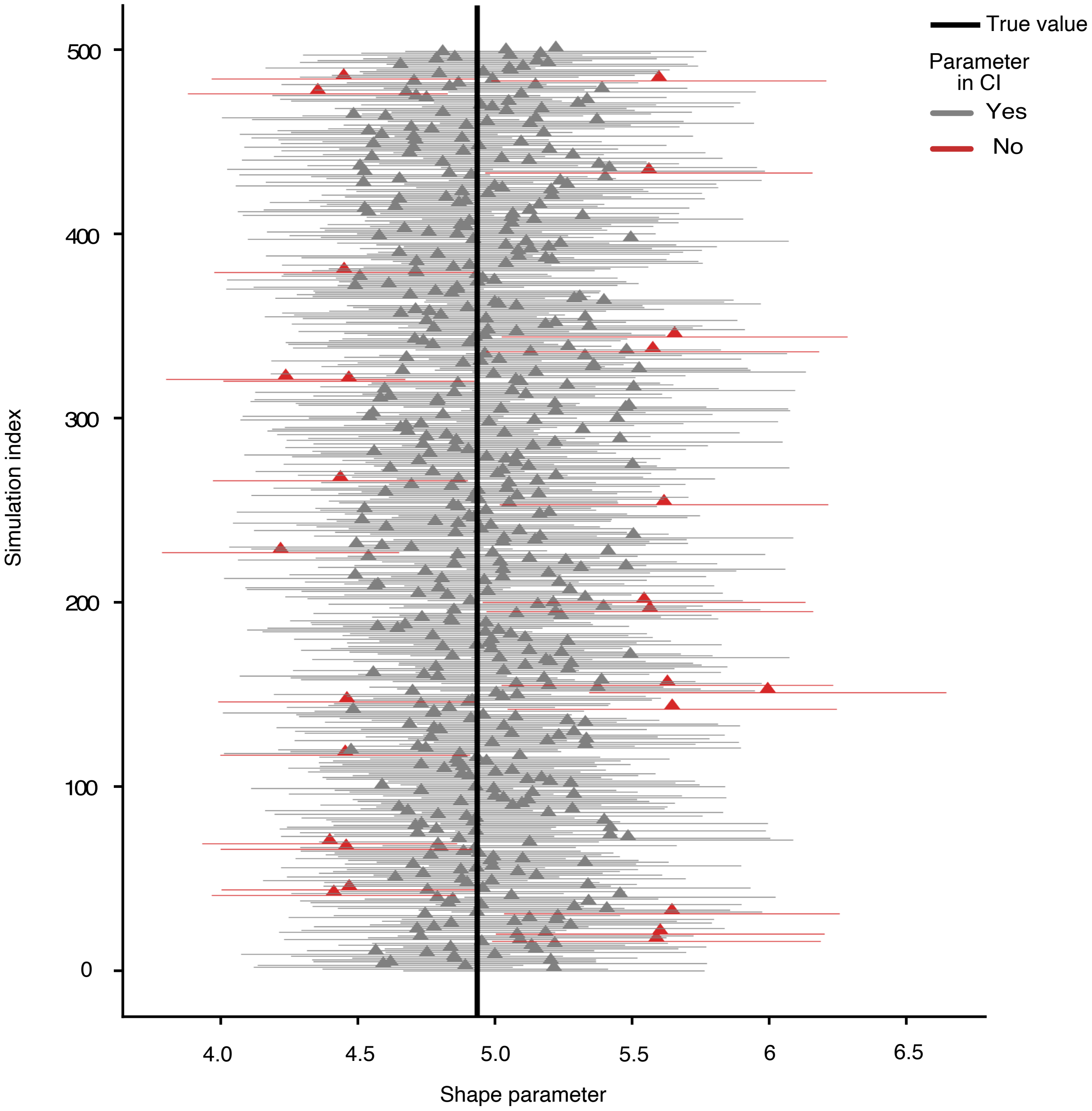

Supplementary Figure 5: Coverage rate for the ML inference method. Estimates of the fluorescence shape parameter for one genetic construct are plotted with their associated 95% confidence interval. The genetic construct was characterized after simulating a Flow-seq experiment with 1018 genetic variants, 10^7 sequencing reads, 10^6 cells sorted, a PCR amplification factor of 10^2, 8 bins for sorting cells, and maximum fluorescence of $f_{max} = 10^5$, using the fluorescence distribution parameters from the Taniguchi library. 500 different replicate simulations were generated to compute the coverage rate of the confidence interval, which as expected is also 95%.

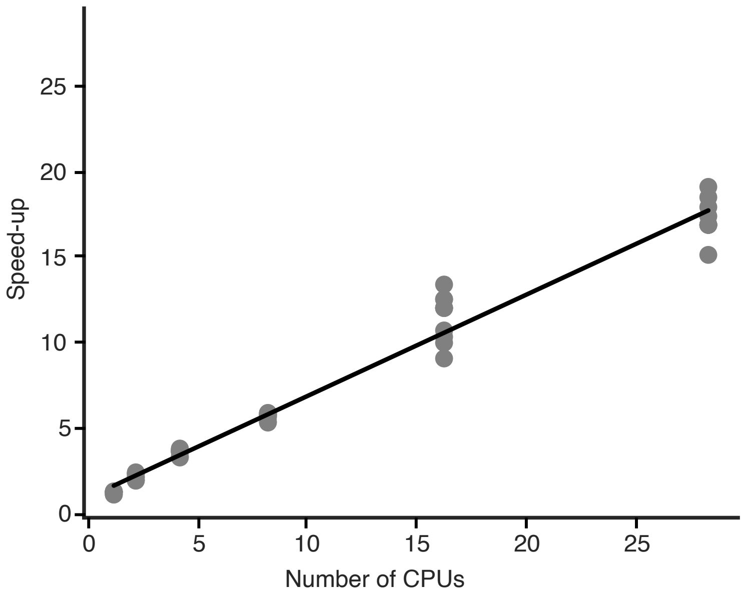

Supplementary Figure 6: Scalability of the parallelized ML estimator. Measuring scaling performance for ML inference to increasing numbers of CPUs. Each point corresponds to an independent measurement. The fitted observed linear speedup (solid black line) has a gradient of 0.6.

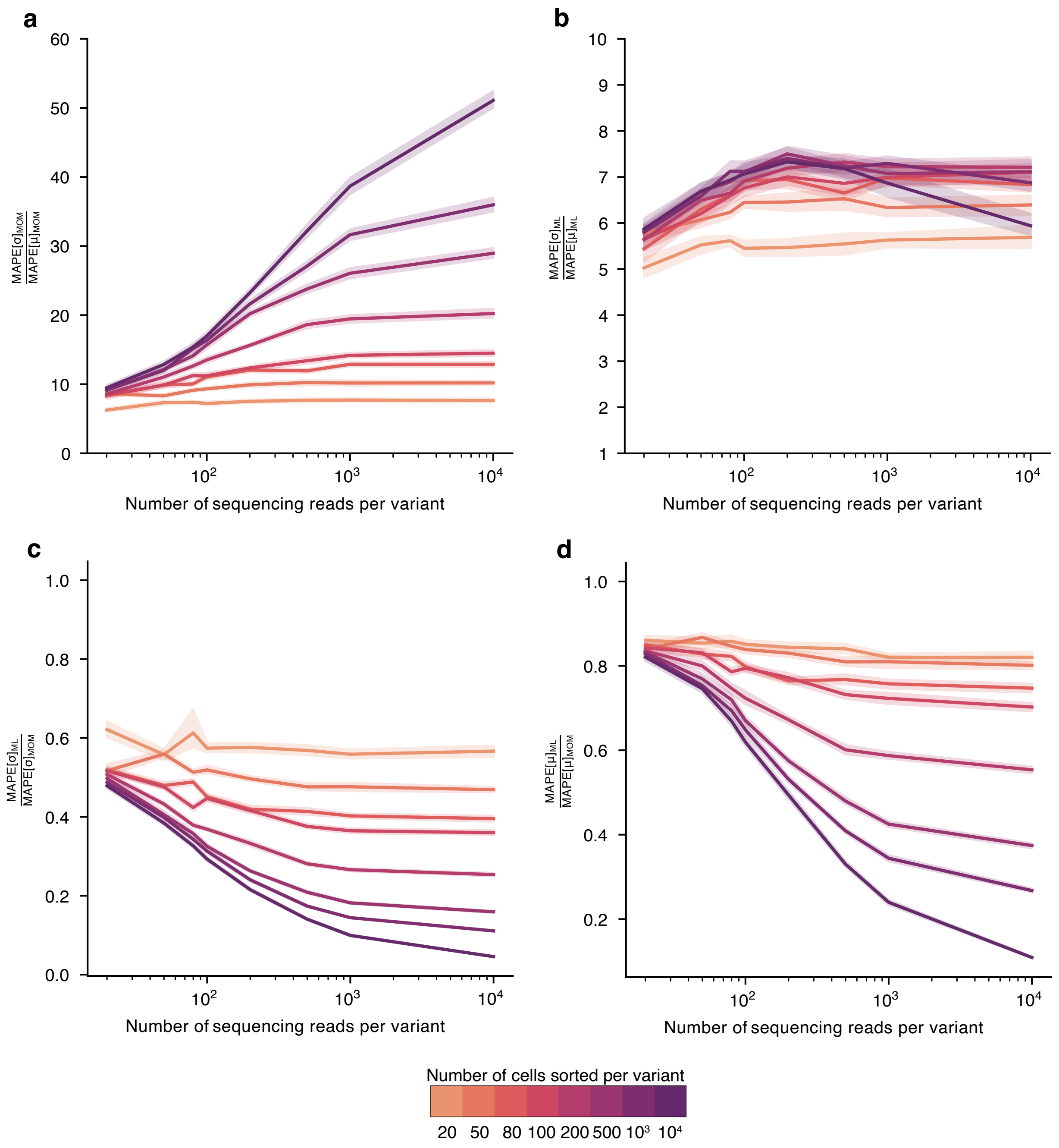

Supplementary Figure 7: Differences in inference accuracy. Estimation of the fluorescence standard deviation is less accurate then estimation of the fluorescence mean. The ratio between the MAPE of the fluorescence standard deviation and the MAPE of the fluorescence mean when using (**a**) MOM inference or (**b**) ML inference is always greater than 1. Inference using ML always yields a smaller relative error than inference using MOM with the ratio between the MAPE using ML and MAPE using MOM always being smaller than 1 when estimating the (**c**) fluorescence standard deviation and (**d**) the fluorescence mean.

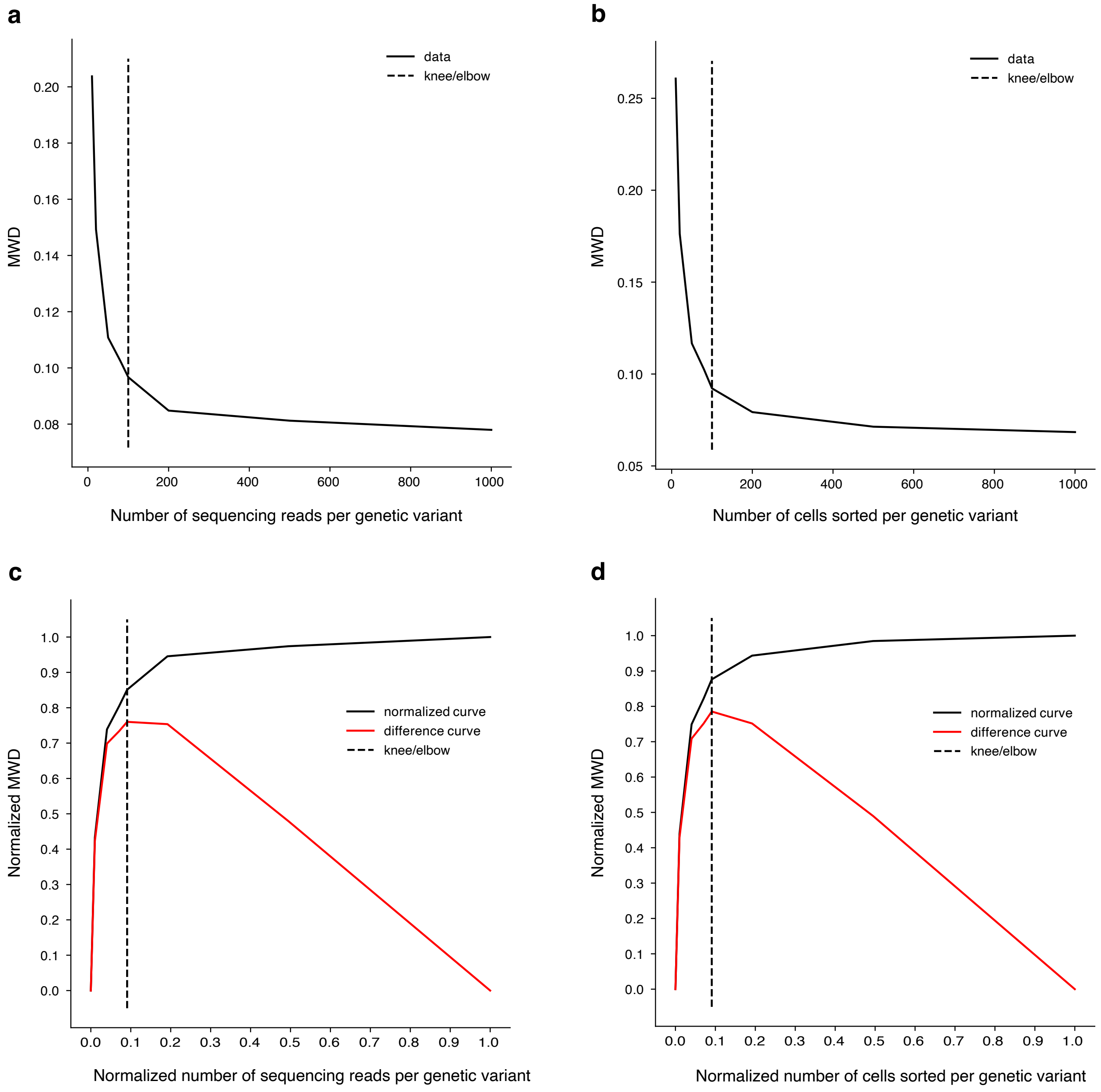

Supplementary Figure 8: Optimal number of cells and sequencing reads when using MOM. (**a**) Mean 1-Wasserstein distance (MWD) between MOM-inferred and ground truth fluorescence distributions as a function of the number of sequencing reads. We picked the simulation with 80 cells per genetic variant during the cell sorting. (**b**) Mean 1-Wasserstein distance (MWD) between MOM-inferred and ground truth fluorescence distributions as a function of the number of cells sorted.We picked the simulation with 80 sequencing reads per genetic variant during the sequencing step. (**c**) Normalised version of panel a. (**d**) Normalised version of panel c. The Mean 1-Wasserstein distance was obtained by averaging over all genetic constructs in the Taniguchi library and all Flow-seq replicates.

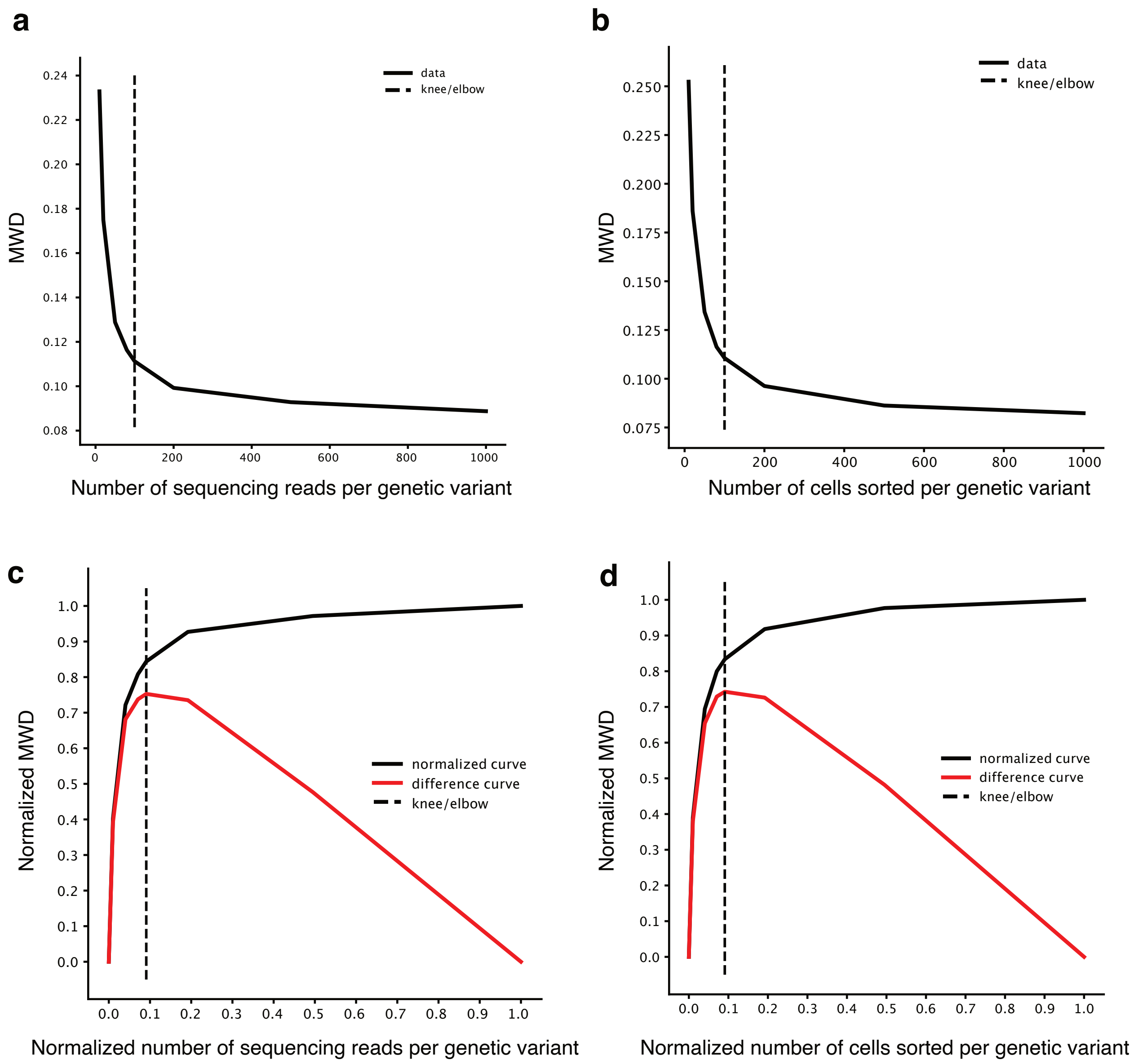

Supplementary Figure 9: Optimal number of cells and sequencing reads when using ML. (**a**) Mean 1-Wasserstein distance (MWD) between ML-inferred and ground truth fluorescence distributions as a function of the number of sequencing reads. We picked the simulation with 80 cells per genetic variant during the cell sorting. (**b**) Mean 1-Wasserstein distance (MWD) between ML-inferred and ground truth fluorescence distributions as a function of the number of cells sorted.We picked the simulation with 80 sequencing reads per genetic variant during the sequencing step. (**c**) Normalised version of panel a. (**d**) Normalised version of panel c. The Mean 1-Wasserstein distance was obtained by averaging over all genetic constructs in the Taniguchi library and all Flow-seq replicates.

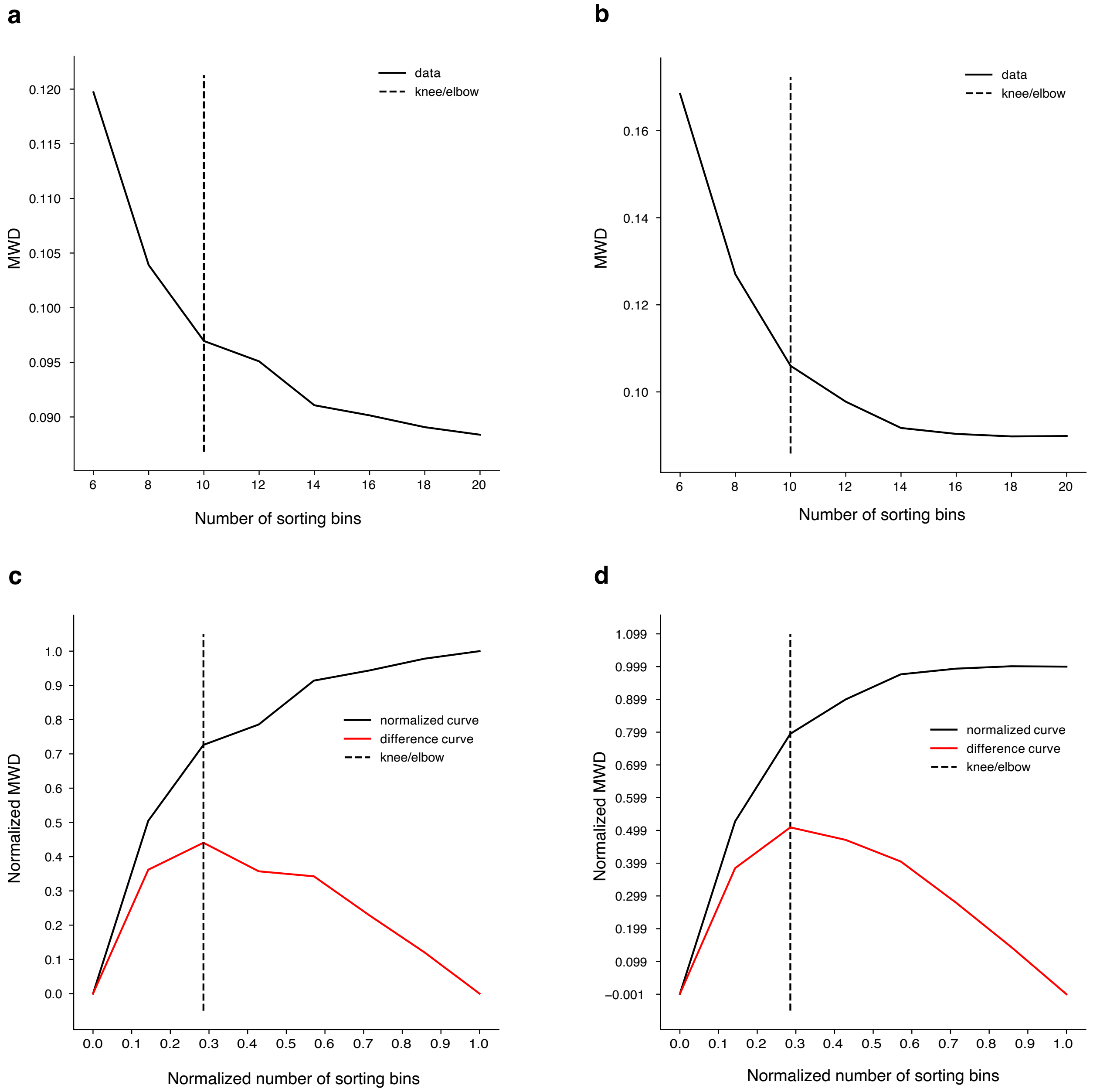

Supplementary Figure 10: Optimal number of bins for cell sorting when using MOM and ML. (**a**) Mean 1-Wasserstein distance (MWD) between ML-inferred and ground truth fluorescence distributions as a function of the number of sorting bins. (**b**) Mean Wasserstein distance between MOM-inferred and ground truth fluorescence distribution as a function of the number of sorting bins. (**c**) Normalised version of panel a. (**d**) Normalised version of panel b. The Mean 1-Wasserstein distance was obtained by averaging over all genetic constructs in the Taniguchi library and all Flow-seq replicates. We performed the simulation with the optimal number of sequencing reads (100 reads per genetic variant) and sorted cells (100 cells per genetic variant).

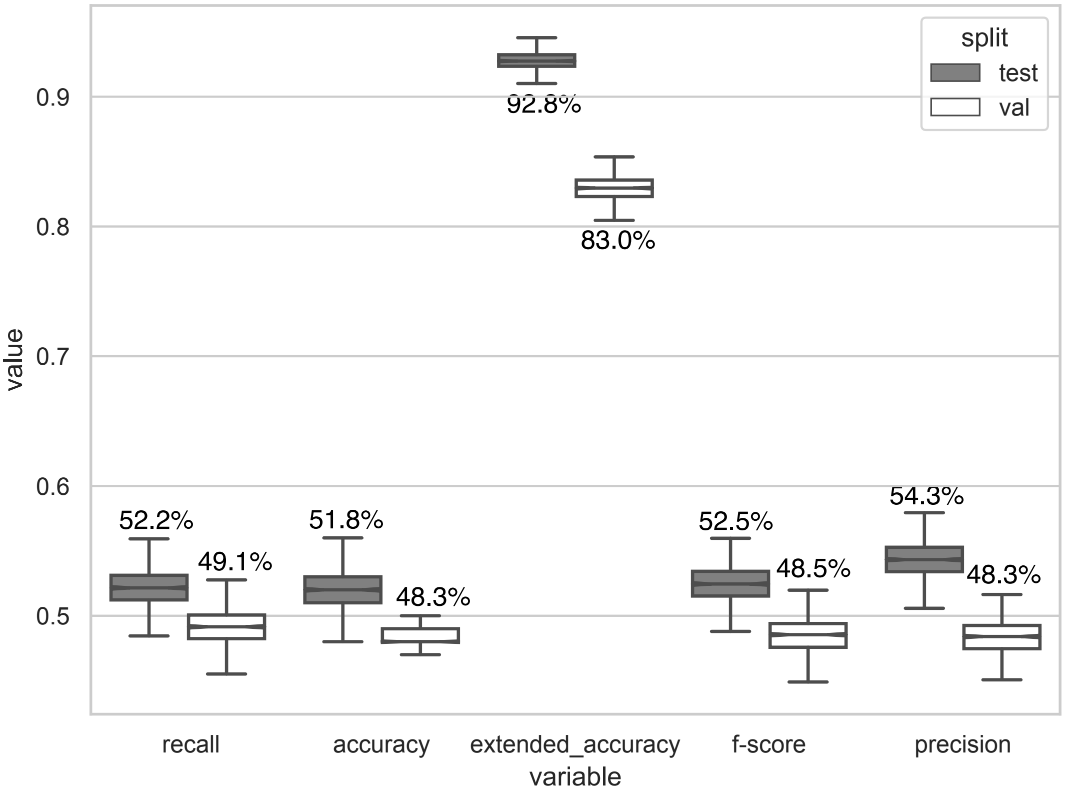

Supplementary Figure 11: Evaluating the hybrid CNN+RNN neural network performance. Performance across key metrics on the validation (white) and test (grey) datasets. Extended accuracy refers to the accuracy of the mode bin prediction allowing for a single bin deviation (i.e., allowing for incorrect predictions if they fall in a neighbouring bin). 95% confidence intervals were computed using 1000 bootstrap samples from each dataset.

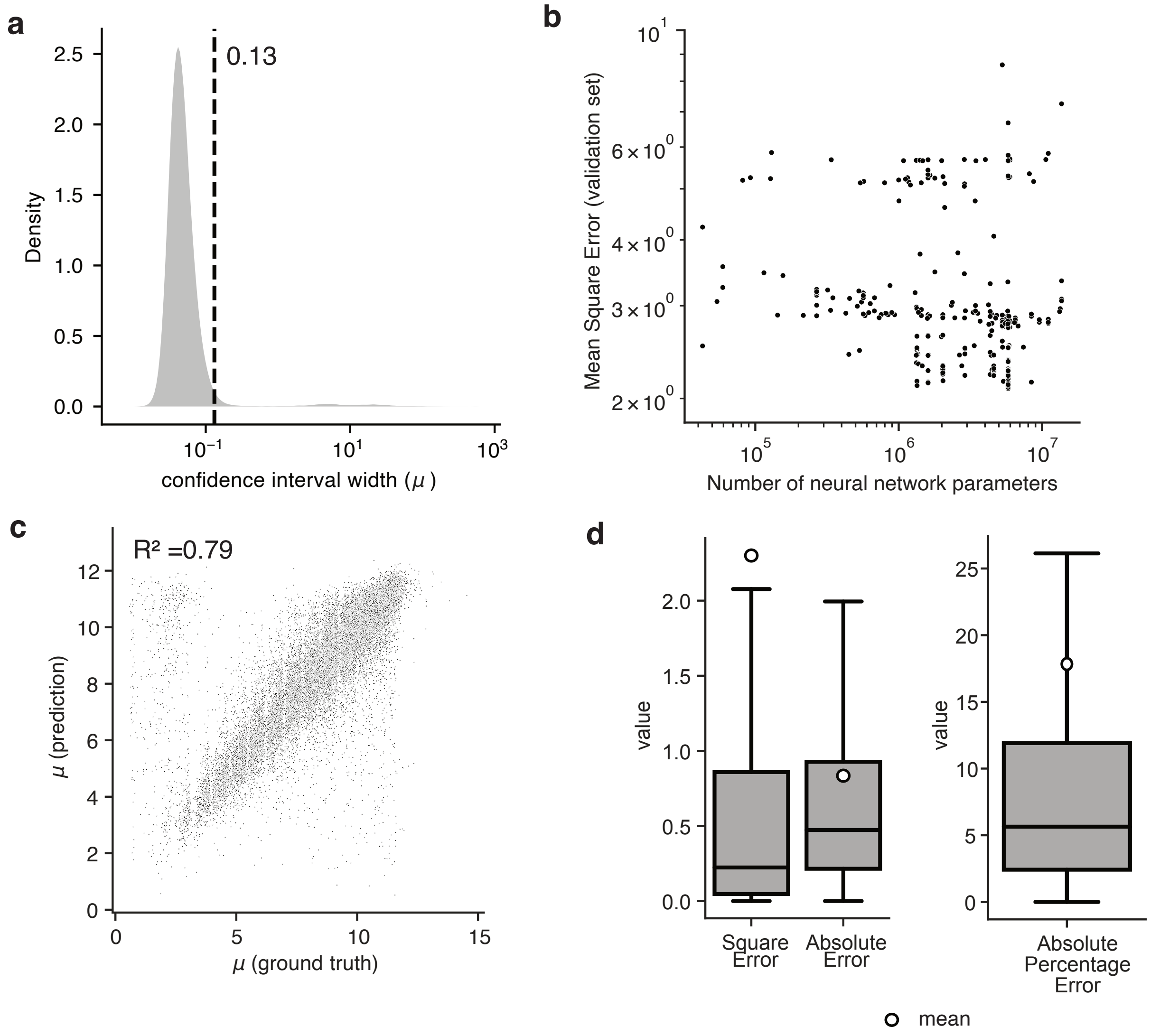

Supplementary Figure 12: Accuracy limits for models trained on the Cambray *et al.* data set. (**a**) Distribution of the 99.7% confidence interval width of the mean log-fluorescence μ computed using maximum likelihood across sequences from the Cambray dataset. Dashed line indicates the median 99.7 % confidence interval width (.13) (**b**) Number of parameters and best mean square error (evaluated on the validation set) of all hybrid CNN-BiLSTM models considered during the hyperparameter optimization step on the Cambray data. (**c**) Test set predictions of the best hybrid CNN-BiLSTM model predicting the mean log-fluorescence. (**d**) Distribution of the test set performance metrics of the best hybrid CNN-BiLSTM model.

REFERENCES

[1] Shahmohammadi, A. & McAuley, K. B. Sequential Model-Based A-Optimal Design of Experiments When the Fisher Information Matrix Is Noninvertible. *Industrial & Engineering Chemistry Research* **58**, 1244–1261 (2019).

[2] Pauwels, E., Lajaunie, C. & Vert, J.-P. A Bayesian active learning strategy for sequential experimental design in systems biology. *BMC Systems Biology* **8**, 102 (2014).

[3] Beal, J. *et al.* Meeting Measurement Precision Requirements for Effective Engineering of Genetic Regulatory Networks. *ACS Synthetic Biology* **11**, 1196–1207 (2022).

[4] Robert, C. P. *The Bayesian Choice: From Decision-Theoretic Foundations to Computational Implementation*. Springer Texts in Statistics (Springer, New York, 2007), 2nd ed edn.

[5] Taniguchi, Y. *et al.* Quantifying E. coli Proteome and Transcriptome with Single-Molecule Sensitivity in Single Cells. *Science* **329**, 533–538 (2010).

[6] Cambray, G., Guimaraes, J. C. & Arkin, A. P. Evaluation of 244,000 synthetic sequences reveals design principles to optimize translation in *Escherichia coli*. *Nature Biotechnology* **36**, 1005–1015 (2018).
